# Quantification of human mature frataxin protein expression in nonhuman primate hearts after gene therapy

**DOI:** 10.21203/rs.3.rs-3121549/v1

**Published:** 2023-06-29

**Authors:** Ian Blair, Teerapat Rojsajjakul, Juliette Hordeaux, Gourav Chaudhary, Christian Hinderer, Clementina Mesaros, James Wilson

**Affiliations:** University of Pennsylvania; University of Pennsylvania; University of Pennsylvania; University of Pennsylvania; University of Pennsylvania; Perelman School of Medicine, University of Pennsylvania; University of Pennsylvania

## Abstract

Deficiency in human mature frataxin (hFXN-M) protein is responsible for the devastating neurodegenerative and cardiodegenerative disease of Friedreich’s ataxia (FRDA). It results primarily by epigenetic silencing the FXN gene due to up to 1400 GAA triplet repeats in intron 1 of both alleles of the gene; a subset of approximately 3% of FRDA patients have a mutation on one allele. FRDA patients die most commonly in their 30s from heart disease. Therefore, increasing expression of heart hFXN-M using gene therapy offers a way to prevent early mortality in FRDA. We used rhesus macaque monkeys to test the pharmacology of an adeno-associated virus (AAV)hu68.CB7.hFXN therapy. The advantage of using non-human primates for hFXN-M gene therapy studies is that hFXN-M and monkey FXN-M (mFXN-M) are 98.5% identical, which limits potential immunologic side-effects. However, this presented a formidable bioanalytical challenge in quantification of proteins with almost identical sequences. This was overcome by development of a species-specific quantitative mass spectrometry-based method, which revealed for the first time, robust transgene-specific human protein expression in monkey heart tissue. The dose response was non-linear resulting in a ten-fold increase in monkey heart hFXN-M protein expression with only a three-fold increase in dose of the vector.

## Introduction

**Figure 1:**
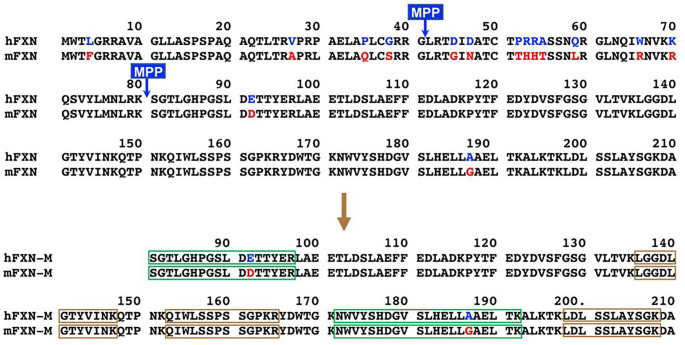
Formation of hFXN-M (81–210, upper) and Resus macaque mFXN-M (lower, 81–210). Full length hFXN (1–210) and full-length mFXN (1–210) translocate to the mitochondria where both hFXN-M (81–210, upper) and mFXN-M (81–210, lower) are formed as 130 amino acid proteins by sequential mitochondrial processing peptidase (MPP)-mediated cleavage at G^41^-L^42^ and K^80^-S^81^. Amino acid differences are shown in blue (human) and red (monkey). The two tryptic peptides that were quantified to differentiate hFXN-M from mFXN-M are shown in green boxes whereas, three common tryptic peptides that were analyzed as qualifying peptides are shown in brown boxes.

Friedreich’s ataxia (FRDA) is a neurodegenerative and cardiodegenerative autosomal recessive genetic disease resulting from an intronic GAA triplet repeat expansion in the *FXN* gene^1,2^. FRDA has a prevalence of 1 in 50,000–100,000 individuals in the USA, and so is the most common hereditary ataxia^3^. This devastating disease, which is characterized by ataxia and other neurological defects^3^, arises from a deficiency in human mature frataxin (hFXN-M), a 130 amino acid mitochondrial protein^4^. Full length hFXN protein (1–210, Fig. 1) expressed in the cytosol, translocates to the mitochondria where it undergoes sequential mitochondrial processing peptidase (MPP) cleavage at G^41^-L^42^ and K^80^-S^81^ to produce hFXN-M protein (Fig. 1)^5,6^. Mitochondrial hFXN-M protein plays an important role in the biogenesis and maintenance of Fe-S clusters and in persulfide processing^7–10^. Epigenetic silencing of the *FXN* gene due to the presence of up to 1400 GAA triplet repeats in intron1 of both alleles of the *FXN* gene (homozygous patients) results in reduced transcription of *FXN* mRNA, reduced expression of full length hFXN (1–210) in the cytosol, and reduced amounts of hFXN-M produced in the mitochondria^1,11^. A subset of approximately 3% of FRDA patients have a mutation on one allele and GAA repeats in intron 1 of the other allele (complex heterozygotes)^1,4^. The presence of mutated proteins that could potentially be expressed in complex heterozygous patients has not been reported, suggesting that the small amount of transcribed hFXN-M protein comes from the allele containing GAA repeats in intron 1. Symptoms of FRDA generally appear during adolescence; patients slowly progress to wheelchair dependency within 15 years and patients die most commonly in their 30s from heart disease^3^. Expression of hFXN-M decreases with increased GAA repeat length causing an earlier age of onset and increased in disease severity^12,13^. Therefore, increasing expression of hFXN-M in the heart of both homozygous and complex heterozygous patients using gene therapy offers a potential route to preventing early mortality resulting from cardiac failure.

Studies on gene therapy using rodent models of FRDA have provided encouragement for this approach as well as highlighting the need for caution^14^. For gene delivery, the most common approach has been the use of adeno-associated virus (AAVs) because the gene delivered by this vector does not integrate into the patient genome and has a low immunogenicity^15^. In addition, the potential of these vectors has been established by numerous preclinical and clinical studies, as well as by already approved therapies^15^. Intravenous systemic delivery of the gene is the most widely used method because of its extremely low invasiveness^14^. This approach was used in the conditional Mck mouse model of FRDA where there is complete deletion of the *FXN* gene in cardiac and skeletal muscle^16^. Intravenous administration by the retro-orbital route with AAVrh10 expressing hFXN-M (5.4E13 vector genomes/kg) resulted in the vector being readily transported to the myocardium where hFXN-M expression prevented the onset of cardiac disease. Furthermore, later administration of the same amount of AAVrh10 vector after the onset of heart failure, was able to completely reverse the cardiomyopathy of the mice at the functional, cellular, and molecular levels^16^. A subsequent study was conducted in a mouse model with partial cardiac-specific excision of *FXN* exon 4 in the heart using the Cre-Lox recombination^17^. This FRDA cardiac-specific mouse model has a mild phenotype like the early human clinical cardiomyopathy of FRDA where the clinical cardiac phenotype requires stress. A single intravenous administration of AAVrh.10hFXN (1.00E11 vector genomes) was found to relieve the phenotypic outcomes of cardiomyopathy in this cardiac-specific FRDA mouse model^17^.

It has been reported that hFXN-M cardiac overexpression up to 9-fold the normal endogenous mouse FXN-M proteoform levels in mice was safe, but significant toxicity to the heart at levels above 20-fold was found^18^. However, the methodology that was used could not distinguish between mouse FXN-M proteforms, hFXN-M, or hFXN-M that was processed in the mouse hearts. Therefore, little is known about the relationship between the dose of hFXN-M vector and the expression of the hFXN-M protein in target tissue. To address this important issue, we have used rhesus macaque monkeys as a non-human primate model because hFXN-M and monkey FXN-M (mFXN-M) are 98.5% identical (Fig. 1). Using a highly specific stable isotope dilution immunoaffinity precipitation ultra-high performance liquid chromatography-multiple reaction monitoring mass spectrometry (IP-UHPLC-MRM/MS) method, we have quantified both hFXN-M and mFXN-M in Rhesus macaque monkeys 1-month after they were treated with escalating doses of an AAVhu68 clade F vector expression hFXN-M under the ubiquitous promoter CB7.

## Results

### IP-UHPLC-MRM/MS analysis of control heart tissues

**Fig. 2:**
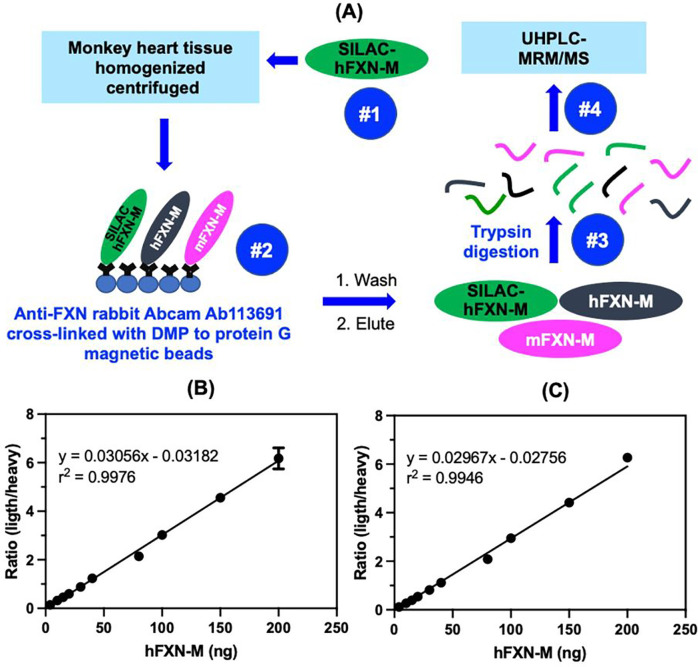
Workflow for quantification of hFXN-M and mFXN-M. (A) Schematic showing workflow for quantification of hFXN and mFXN-M. (B) Typical standard curve for SGTLGHPGSLD**E**TTYER tryptic peptide used for quantitative analysis of analysis of hFXN-M and mFXN-M in monkey heart. (C) Typical standard curve for KNWVYSHDGVSLHELL**A**AELTKALKTKLDL tryptic peptide for quantitative analysis of analysis of hFXN-M and mFXN-M in monkey heart.

**Fig. 3:**
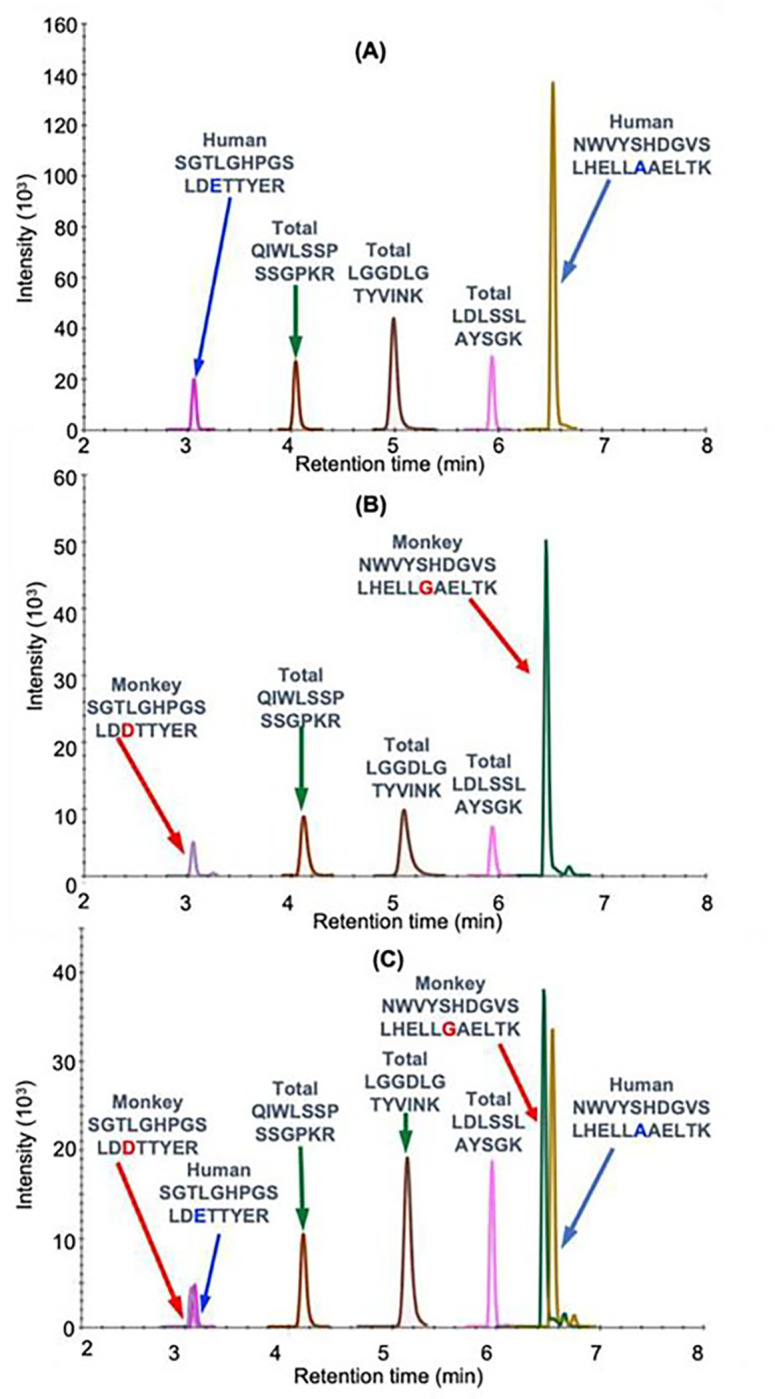
UHPLC-MR/MS analysis of FXN-M tryptic peptides (A) hFXN-M from human heart. (B) mFXN-M from Rhesus macaque heart. (C) 50:50 mixture of hFXN-M and mFXN-M. The most intense MRM transitions for each peptide were used: SGTLGHPGSLD**D**TTYER$\left({y_{14}}^{2+}\right)$, SGTLGHPGSLD**E**TTYER $\left({y_{14}}^{2+}\right)$, QIWLSSPSSGPKR $\left({y_{8}}^{+}\right)$, LGGDLGTYVINK $\left({y_{10}}^{+}\right)$, LDLSSLAYSGK $\left({y_{10}}^{+}\right)$, NWVYSHDGVSLHELL**G**AELTK $\left({y_{19}}^{3+}\right)$, NWVYSHDGVSLHELL**A**AELTK$\left({y_{19}}^{3+}\right)$.

Bioactive proteins are found in heart tissue in the presence of high abundance proteins (HAPs) such as vimentin and myosin that are often 10^6^ to 10^7^ higher in concentration. Simple extraction procedures result interference from the HAPs as well as suppression of the MS signal from the target protein by the HAPs. Removal of the HAPs by immunodepletion can cause the loss of the target protein through non-covalent binding to them^19^. Low abundance proteins such hFXN-M and mFXN-M can also be lost during the extraction of the biofluid or tissue through non-covalent binding to glassware and plastic surfaces as we have shown previously for amyloid-β proteins^20^. Immunoprecipitation (IP) can be used to purify target proteins, but recovery from different heart tissue samples can be inconsistent. Digestion by proteases such as trypsin, which is required to generate peptides that are amenable to specific and sensitive quantification by UHPLC-MRM/MS (Fig. 2A), can also be inconsistent for different tissue samples. UHPLC-MRM/MS is not a quantitative tool because differential ionization of peptides can occur in the source of the mass spectrometer. Therefore, a heavy isotope internal protein standard prepared using stable isotope labeling by amino acids in cell culture (SILAC), is added to the heart tissue sample at the start of the isolation procedure (Fig. 2A). The SILAC standard acts as a carrier to prevent losses during isolation, as an internal control to normalize extraction and protease digestion efficiency, and to compensate for differential ionization in the mass spectrometer source. This permits accurate and precise protein quantification to be conducted^13^. In the present study, homogenized frozen control (non-FRDA) human and rhesus macaque monkey heart tissues (25 mg to 150 mg) spiked with SILAC-hFXN-M (40 ng) were purified by IP (Fig. 2A). The IP removed most of the interfering proteins and the SILAC-hFXN-M provided an internal control that compensated for any losses during the procedure. Protease digestion of the hFXN-M and mFXN-M in the IP eluate with trypsin provided numerous of peptides from each protein (Fig. 3). The identity of hFXN-M (Fig. 3A) and mFXN-M (Fig. 3B) was established by the presence of 5 tryptic peptides from each protein in the relevant UHPLC-MRM/MS chromatogram. The use of three MRM transitions for each of the peptides provided the specificity that made it possible to unequivocally differentiate the two proteins. There are two amino acid differences between hFXN-M and mFXN-N (**E**92**D** and **A**187**G**) in tryptic peptides S^81^GTLGHPGSLD**E**TTYER^97^, N^172^WVYSHDGVSLHELL**A**AELTK^192^ (hFXN-M) and S^81^GTLGHPGSLD**D**TTYER^97^, N^172^WVYSHDGVSLHELL**G**AELTK^192^ (mFXN-M) (Fig. 1). Therefore, 7 tryptic peptides were observed in the UHPLC-MRM/MS chromatogram when a 50:50 mixture of the two proteins was digested with trypsin (Fig. 3C).

Standard curves prepared in bovine serum albumin surrogate matrix that had been subjected to the same IP-LC-MRRM/MS procedure were linear (Figs. 2B and 2C). Back-calculated amounts of hFXN-M from standard curve samples (n=5) for SGTLGHPGSLD**E**TTYER (Fig. 2B) and NWVYSHDGVSLHELL**A**AELTK (Fig. 2C) hFXN-M had a precision of better than 15% and accuracy of between. 85% and 115% (Tables 2A and 2B). UHPLC-MRM/MS was conducted using the transitions shown for the light tryptic (unlabeled) peptides shown in Table 1. A chromatogram of the most abundant UHPLC-MRM/MS tryptic peptide transitions from human heart revealed an excellent separation of the five peptides from hFXN-M. The amino terminal SGTLGHPGSLD**E**TTYER tryptic peptide, which distinguished hFXN-M from mFXN-M by virtue of **E**-92 instead of **D**-92 was the most polar peptide and eluted at 3.05-min. The least polar peptide NWVYSHDGVSLHELL**A**AELTKALK, which distinguished hFXN-M from mFXN-M by virtue of a **A**-187 instead of **G**-187 eluted at 6.56 min. A chromatogram of the most abundant UHPLC-MRM/MS transitions of mFXN tryptic peptides from monkey heart also revealed that there was excellent separation of the five tryptic peptides from mFXN-M (Fig. 3B). The amino SGTLGHPGSLD**D**TTYER tryptic peptide, was the most polar peptide and eluted at 3.03-min and he least polar peptide NWVYSHDGVSLHELL**G**AELTKALK, eluted at 6.44 min (Fig. 3B). A chromatogram from a 50:50 mixture of hFXN-M from human heart and mFXN-M from monkey heart showed that the two tryptic peptides with different amino acid sequences (SGTLGHPGSLD**E**TTYER and SGTLGHPGSLD**D**TTYER, NWVYSHDGVSLHELL**A**AELTKALK and NWVYSHDGVSLHELL**G**AELTKALK) could be separated from each other so that hFXN-M and mFXN-M proteins could be readily distinguished (Fig. 3C). The two unique tryptic peptides from hFXN and mFXN were separated either through their different MRM transitions (N-terminal SGT peptides) or by a combination of their different MRM transitions and different retention times (NWV-peptides) (Table 1).

### Stable isotope dilution IP-UHPLC-MRM/MS quantitative analysis of control (non-FRDA) heart tissues

Many quantitative MS-based studies of protein expression rely on the use of isotopically stable labeled peptide (AQUA) standards. AQUA standards give excellent precision because they compensate for differences in the ionization efficiency in the mass spectrometer. However, they have poor accuracy, particularly when IP is used for protein isolation because they do not take account of losses during the procedure. They also do not take account of inter-sample differences in the efficiency of protein digestion as we showed for apolipoprotein (Apo)A1 protein in human serum^21^. Addition of an AQUA peptide prior to protease digestion results in differential loss of the peptide during digestion when compared with the protein-derived peptide^22^ and so this approach cannot be used. These problems can be readily overcome using SILAC protein internal standards as we have demonstrated for amyloid-β proteins in cerebrospinal fluid (CSF)^20^, apolipoprotein-A1 in serum^21^, hFXN-M and hFXN-E in whole blood^13^, high mobility group box1 (HMGB1) in human plasma and serum^23^, oxidized HMGB1 in cell media^24^, and mouse FXN-M in mouse heart, brain, and liver tissues^25^. The ratio between the SILAC protein the corresponding endogenous protein is established at the start of the isolation procedure. This ratio remains the same throughout the entire procedure and is used to calculate the amount of endogenous protein from a standard curve that is constructed at the same time with an authentic protein standard. The SILAC protein also serves as a carrier to enhance the recovery of low-level tissue proteins that can be lost through non-selective binding to glassware and plastic surfaces. This was unequivocally demonstrated in our assay for amyloid-β proteins in CSF^20^, where the proteins are almost completely lost in the absence of a stable isotope carrier through binding to surfaces during isolation and analysis. Therefore, we employed SILAC-hFXN-M as the internal standard for quantifying both hFXN-M and mFXN-M in monkey heart.

**Fig. 4:**
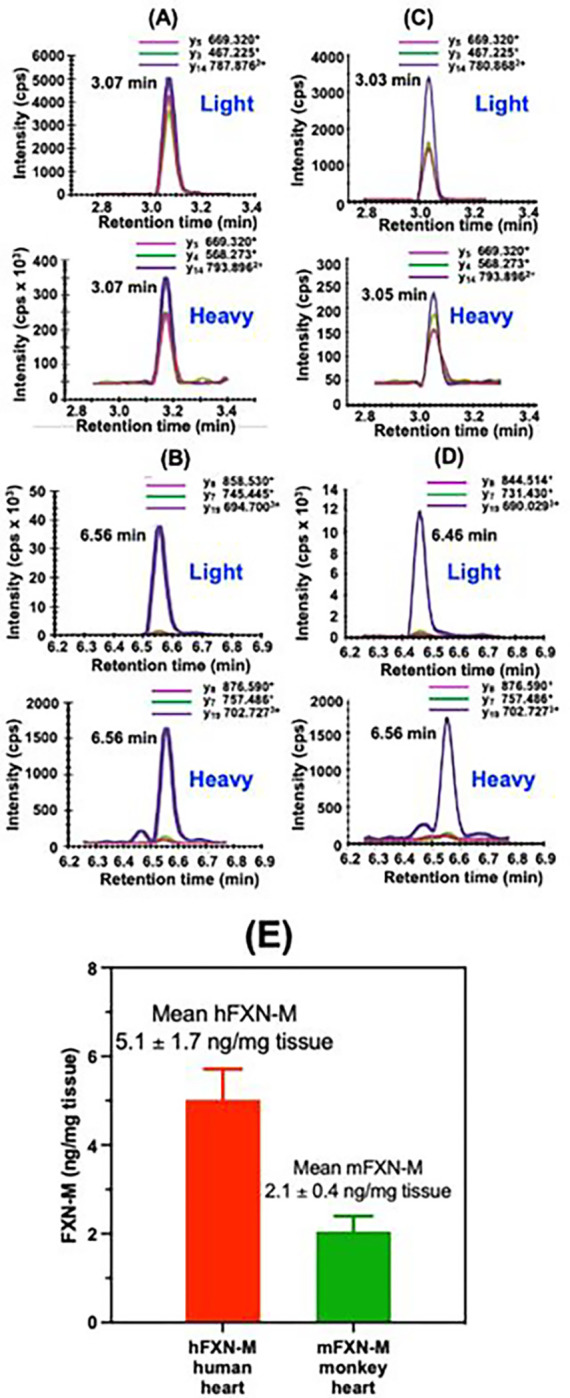
Typical UHPLC-MRM/MS chromatograms of tryptic peptides for quantification of hFXN-M (4.8 ng/mg tissue) from a control (non-FRDA) human heart and mFXN-M (1.9 ng/mg tissue) from a control Resus macaque monkey heart. (A) SGTLGHPGSLD**E**TTYER tryptic peptide from hFXN-M (${{\text{MH}}_{3}}^{3+}=607.287$; upper) and heavy SGT**L**GHPGS**L**D**E**TTYER internal standard (${{\text{MH}}_{3}}^{3+}=611.300$; lower). (B) NWVYSHDGVSLHELL**A**AELTK tryptic peptide from hFXN-M (${{\text{MH}}_{4}}^{4+}=596.308$; upper) and heavy NWVYSHDGVS**L**HE**LL****A**AE**L**TK internal standard (${{\text{MH}}_{4}}^{4+}=602.328$; lower). (C) SGTLGHPGSLD**D**TTYER tryptic peptide from mFXN-M (${{\text{MH}}_{3}}^{3+}=602.615$; upper) and heavy SGT**L**GHPGSLDETTYER internal standard (${{\text{MH}}_{3}}^{3+}=611.300$; lower). (D) NWVYSHDGVSLHELL**G**AELTK tryptic peptide from mFXN-M (${{\text{MH}}_{4}}^{4+}=592.804$; upper) and heavy NWVYSHDGVS**L**HE**LL****A**AELTK internal standard (${{\text{MH}}_{4}}^{4+}=602.328$; lower). (E) Levels of hFXN-M in control (non-FRDA) human heart tissue (mean ± SD; n=5) and mFXN-M in Rhesus macaque heart tissue from untreated animals (mean ± SD; n=5). **L** = ^13^C_6_-leucine.

Typical UHPLC-MRM/MS chromatograms for SGTLGHPGSLD**E**TTYER tryptic peptide from control (non-FRDA) human heart (upper) and heavy SGTLGHPGSLD**E**TTYER internal standard (lower) are shown in Fig. 4A. Chromatograms for NWVYSHDGVSLHELL**A**AELTK tryptic peptide from control (non-FRDA) human heart (upper) and heavy NWVYSHDGVSLHELL**A**AELTK internal standard (lower) are shown in Fig. 4B. The amount of hFXN-M was then determined for each peptide from the relevant standard curve (Figs. 2B and 2C). The mean level of hFXN-M (4.8 ng/mg tissue) was then calculated from the mean of the two hFXN-M and mFXN-M peptides. Typical UHPLC-MRM/MS chromatograms for SGTLGHPGSLD**D**TTYER tryptic peptide from control monkey heart (upper) and heavy SGTLGHPGSLD**E**TTYER internal standard (lower) are shown in Fig. 4C. Chromatograms for NWVYSHDGVSLHELL**G**AELTK tryptic peptide from control monkey heart (upper) and heavy NWVYSHDGVSLHELL**A**AELTK internal standard (lower) are shown in Fig. 4D. The ratio of sum of the three light peptide MRM transitions to the sum of the three heavy peptide transitions were determined. The amount of each peptide was then determined for each peptide from the relevant standard curve (Figs. 2B and 2C). The mean level of mFXN-M (1.9 ng/mg tissue) was then calculated from the mean of the two mFXN-M peptides. Levels of hFXN-M in five different control (non-FRDA) human heart tissues were determined to be 5.1 ± 1.7 ng/mg tissue (mean ± SD; n=5). Levels of mFXN-M in five different Rhesus macaque heart tissue from untreated animals were 2.1 ± 0.4 ng/mg tissue (Fig. 4E).

### All AAV vector doses resulted in hFXN-M expression in monkey heart

**Figure 5:**
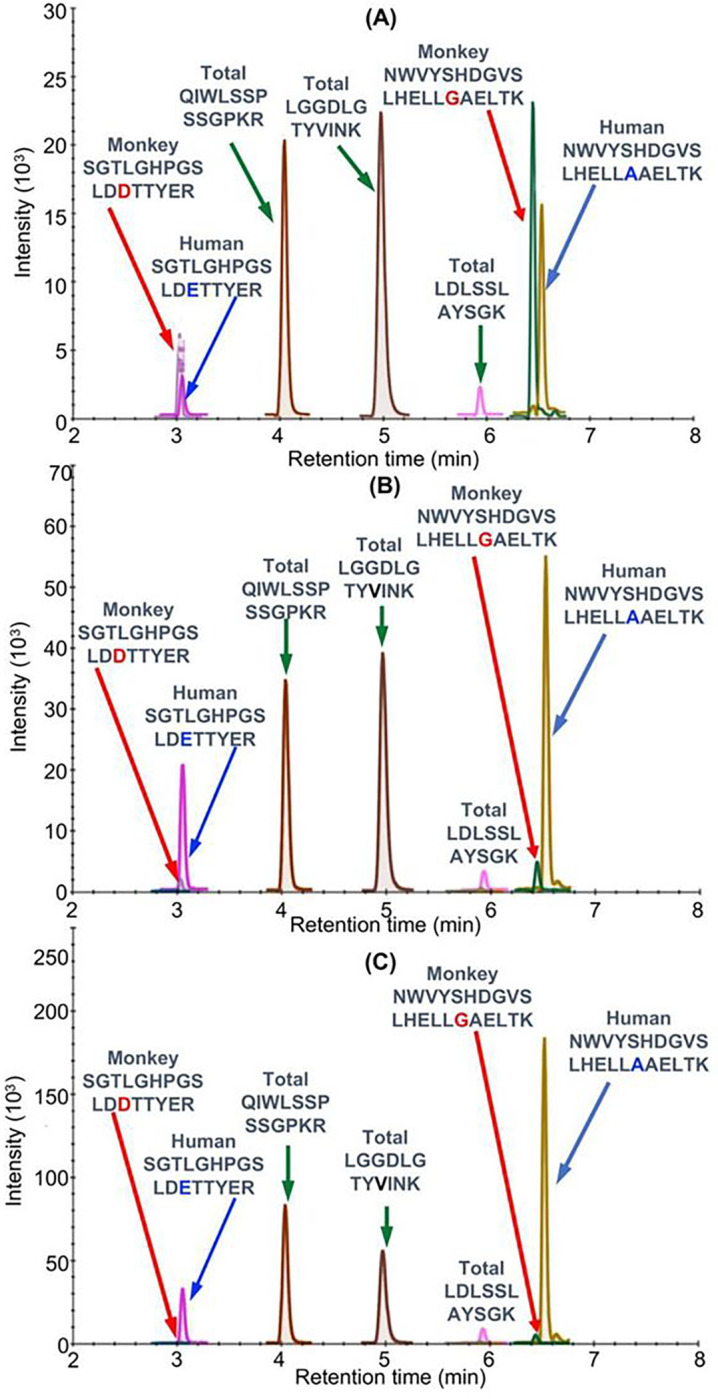
UHPLC-MRM/MS analysis of FXN tryptic peptides in monkey hearts after gene therapy using the same transitions as in legend to Fig. 3. (A) hFXN-M and mFXN-M in the right ventricle of monkey # 2 (RV2 B7, 180616) after gene therapy with the lowest dose of 1.00E13 AAV vector GC/kg. (B) hFXN-M ( 40.1 ng/mg tissue) and mFXN-M (3.4 ng/mg tissue) in the left ventricle of monkey # 3 (LV3 B13, 180701) after gene therapy with the middle dose of 3.00E14 AAV vector GC/kg. (C) hFXN-M (77.0 ng/mg tissue) and mFXN-M (2.3 ng/mg tissue) in the heart septum of monkey # 5 (HS 5, B24, 181271) after gene therapy with the highest dose of AAV vector of 1.00E+14 GC/kg.

UHPLC-MRM/MS chromatograms of monkey tissue after the lowest dose of the AAV vector revealed the presence of the two specific hFXN peptides (SGTLGHPGSLD**E**TTYER and NWVYSHDGVSLHELL**A**AELTKALK) as well as the two specific mFXN peptides (SGTLGHPGSLD**D**TTYER and NWVYSHDGVSLHELL**G**AELTKALK) (Fig. 5A). The three common tryptic peptides (VLTVKLGGDLGTYVINK, QIWLSSPSSGPKRYDWTG, and TKALKTKLDLSSLAYSGK) were also detected (Fig. 5A). UHPLC-MRM/MS chromatograms of monkey tissue after the middle dose of the AAV vector revealed that the two specific hFXN peptides (SGTLGHPGSLD**E**TTYER and NWVYSHDGVSLHELL**A**AELTKALK) were much higher intensity than two specific mFXN peptides (SGTLGHPGSLD**D**TTYER and NWVYSHDGVSLHELL**G**AELTKALK) (Fig. 5B). The three common tryptic peptides (VLTVKLGGDLGTYVINK, QIWLSSPSSGPKRYDWTG, and TKALKTKLDLSSLAYSGK) were also detected (Fig. 5B). UHPLC-MRM/MS chromatograms of monkey tissue after the highest dose of the AAV vector (1.00E14 GC/kg) revealed that the two specific hFXN peptides(SGTLGHPGSLD**E**TTYER and NWVYSHDGVSLHELL**A**AELTKALK) were extremely intense (Fig. 5C). In contrast, the two specific mFXN peptides (SGTLGHPGSLD**D**TTYER and NWVYSHDGVSLHELL**G**AELTKALK) could barely be detected. The three common tryptic peptides (VLTVKLGGDLGTYVINK, QIWLSSPSSGPKRYDWTG, and TKALKTKLDLSSLAYSGK) were also detected (Fig. 5C).

### hFXN-M and mFXN-M levels in monkey heart were comparable after the lowest AAV dose

**Fig. 6:**
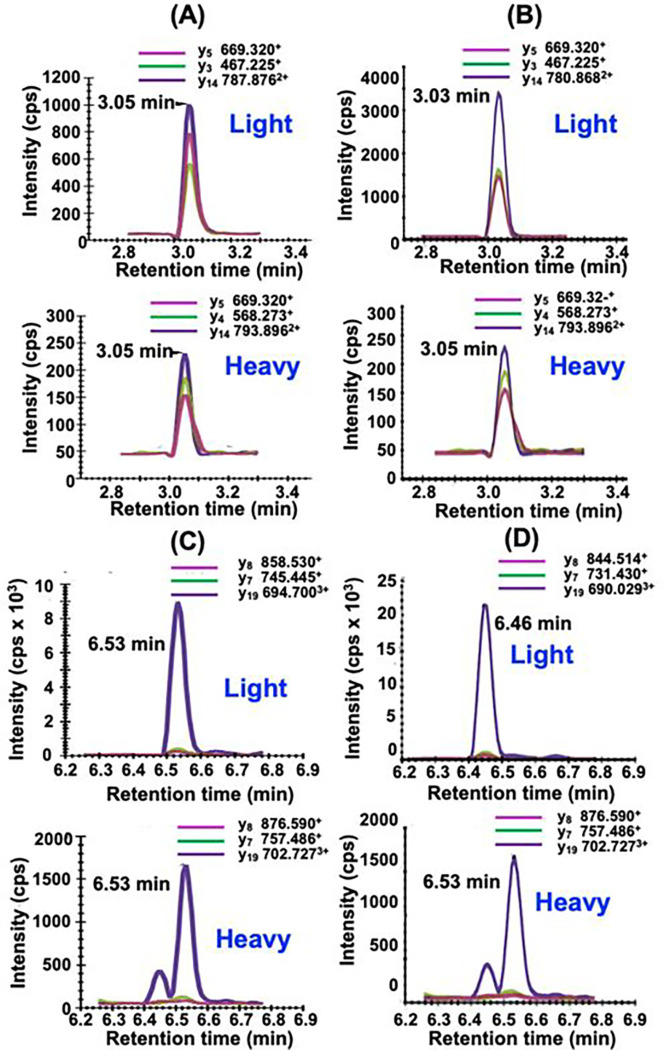
Typical UHPLC-MRM/MS chromatograms of tryptic peptides for quantification of hFXN-M and mFXN-M after lowest vector dose of 1.00E13 GC/kg. (A) SGTLGHPGSLD**E**TTYER tryptic peptide from h-FXN-M (${{\text{MH}}_{3}}^{3+}=607.287$; upper) and heavy SGT**L**GHPGS**L**D**E**TTYER internal standard (${{\text{MH}}_{3}}^{3+}=611.300$; lower). (B) NWVYSHDGVSLHELL**A**AELTK tryptic peptide from hFXN-M (${{\text{MH}}_{4}}^{4+}=596.308$; upper) and heavy NWVYSHDGVS**L**HE**LL****A**AE**L**TK internal standard (${{\text{MH}}_{4}}^{4+}=602.328$; lower). (C) SGTLGHPGSLD**D**TTYER tryptic peptide from mFXN-M (${{\text{MH}}_{3}}^{3+}=602.615$; upper) and heavy SGT**L**GHPGS**L**D**E**TTYER internal standard (${{\text{MH}}_{3}}^{3+}=611.300$; lower). (D) NWVYSHD**G**VSLHELLGAELTK tryptic peptide from mFXN-M (${{\text{MH}}_{4}}^{4+}=592.804$; upper) and heavy NWVYSHDGVS**L**HE**LL****A**AE**L**TK internal standard (${{\text{MH}}_{4}}^{4+}=602.328$; lower). **L** = ^13^C_6_-leucine.

Typical UHPLC-MRM/MS chromatograms of tryptic peptides for quantification of hFXN-M and mFXN-M in monkey heart after lowest dose of 1.00E13 GC/kg of the AAV vector expressing hFXN-M are shown in Fig. 6. Chromatograms for SGTLGHPGSLD**E**TTYER tryptic peptide from human heart (upper) and SILACSGTLGHPGS**L**D**E**TTYER internal standard (lower) are shown in Fig. 6A. Chromatograms for NWVYSHDGVSLHELL**A**AELTK tryptic peptide from human heart (upper) and heavy NWVYSHDGVSLHELL**A**AELTK internal standard (lower) are shown in Fig. 6B. The amount of hFXN-M was then determined for each peptide from the relevant standard curve (Figs. 2B and 2C). The mean level of hFXN-M (1.6 ng/mg tissue) was then calculated from the mean of the two hFXN-M and mFXN-M peptides. Chromatograms for SGTLGHPGSLD**D**TTYER tryptic peptide from control monkey heart (upper) and heavy SGTLGHPGSLD**E**TTYER internal standard (lower) are shown in Fig. 6C. Chromatograms for NWVYSHDGVSLHELL**G**AELTK tryptic peptide from control monkey heart (upper) and heavy NWVYSHDGVSLHELL**A**AELTK internal standard (lower) are shown in Fig. 6D. The ratio of sum of the three light peptide MRM transitions to the sum of the three heavy peptide transitions were determined. The amount of each peptide was then determined for each peptide from the relevant standard curve (Figs. 2B and 2C). The mean level of mFXN-M (3.5 ng/mg tissue) was then calculated from the mean of the two mFXN-M peptides.

### Non-linear hFXN-M expression with increasing dose of AAV vector

**Figure 7:**
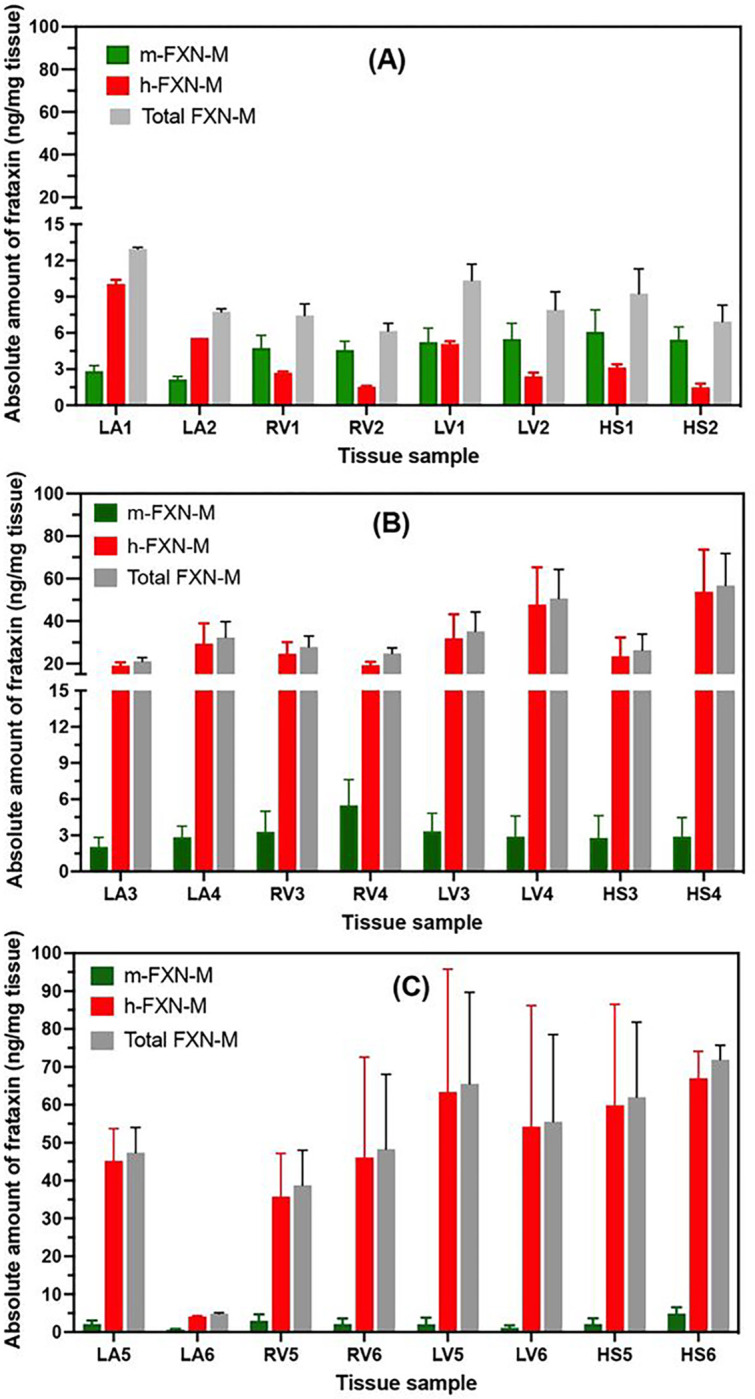
Levels of hFXN-M and mFXN-M in Rhesus macaque heart tissues after IV therapy with increasing doses of AAV. vector. (A). Lowest dose of 1.00+E13 AAV vector genome copies (GC)/kg in two monkeys. (B) Middle dose of 3.00E+13 GC/kg in two monkeys. (C) Highest dose of 1.00E+14 GC/kg in two monkeys. Left atrium = LA; Right ventricle = RV; Left ventricle = LV; Heart septum = HS.

Rhesus macaque heart tissue samples from the left atrium, right ventricle, left ventricle, and septum were analyzed for hFXN-M after the lowest dose of AAV vector (1.00E13 GC/kg). There were no consistent differences in levels in hFXN-M expression from the four different areas of the heart, they varied from 1.5 to 9.8 ng/mg tissue with a mean of 4.1 ± 2.8 ng/mg tissue (Fig. 7A). Similarly, there were no consistent differences in levels in mFXN-M expression from the four different areas of the heart, they varied from 2.4 to 7.9 ng/mg tissue with a mean of 5.6 ± 1.8 ng/mg tissue (Fig. 7A). Therefore, this dose of vector resulted in levels of hFXN-M that were very similar to the levels endogenous mFXN-M. It should however be noted that plateau levels were unlikely reached in this short-term 28-days study. A second analysis of hFXN-M levels in the heart was conducted after the middle dose of AAV vector (3.00E13 GC/kg). There were no consistent differences in levels in hFXN-M expression from the four different areas of the heart, they varied from 20.2 to 67.8 ng/mg tissue with a mean of 37.9 ± 17.6 ng/mg tissue (Fig. 7B). Similarly, there were no consistent differences in levels in mFXN-M expression from the four different areas of the heart, they varied from 2.6 to 7.0 ng/mg tissue with a mean of 4.3 ± 1.3 ng/mg tissue (Fig. 7B). Therefore, this dose of vector resulted in levels of hFXN-M that were 8.8-fold higher than the levels endogenous mFXN-M. Finally, an analysis of hFXN-M levels in heart tissues was conducted after the highest dose of AAV vector (1.00E14 GC/kg). There were no consistent differences in levels in hFXN-M expression from the four different areas of the heart; however, there was one tissue sample where there was very little hFXN-M expression (4.0 ng/mg tissue) (Fig. 7C). The levels varied from 4.0 to 86.3 ng/mg tissue with a mean of 67.7 ± 15.4 ng/mg tissue when the one outlier was excluded (Fig. 7C). Similarly, there were no consistent differences in levels in mFXN-M expression from the four different areas of the heart, they varied from 0.6 to 4.2 ng/mg tissue with a mean of 2.8 ± 1.2 ng/mg tissue (Fig. 7C). Therefore, this dose of vector resulted in levels of hFXN-M that were 24.0-fold higher than the levels of endogenous mFXN-M.

## Discussion

Despite several relevant reports^26–28^ and a non-binding guidance from the US Food and Drug Administration (FDA) on how to conduct long-term gene therapy studies^29^, the pharmacology of gene therapy is still in its infancy. The FDA suggests that long-term plans for monitoring patients who receive gene therapies should be included in study protocols for specific cases described by a decision tree in the guidance^29^. Modeling and pharmacokinetic analysis could significantly aid in the planning for these protocols. The objective of gene therapy is to express DNA or RNA constructs at a desired tissue site *in vivo* tin order to evoke sustained protein expression at levels that can safely achieve a therapeutic effect^27^. To inform dosing decisions, it has been customary to use mRNA levels as a surrogate indicator of tissue protein expression, despite numerous reports that there is a poor relationship between mRNA levels and protein expression in tissues. For example, it was found that in a xenograft model system differentially expressed mRNAs correlated significantly better with the levels of expressed protein than non-differentially expressed mRNAs^30^. Another study showed that that protein concentrations correlated with the corresponding mRNA levels by only 20–40%, and that mRNA abundances were poor predictors of protein expression levels^31^. Furthermore, the relationship between mRNA levels and protein expression in T-cells can be gene class specific and is associated with particular amino acid sequence characteristics^32^. Protein levels are also affected by different conditions such as whether tissues are in steady state, undergoing long-term state changes or subjected to acute perturbations^33^. Considering there is a documented poor relationship between differential mRNA and protein expression, there is a need for a reliable and direct method to quantify tissue protein expression to authoritatively inform gene therapy dose selection decisions.

Several previous studies have addressed the relationship between gene therapy and expressed protein levels. For example, it was reported that a single dose of AAV gene therapy resulted in sustained serum levels of immunotherapeutic proteins^34^. The study reported that the gene therapy resulted in sustained protein expression in the systemic circulation for up to 1 year in mice. Another study addressed the use of AAV gene therapy for systemic protein delivery, where it was suggested that further investigation into AAV expression at the tissue level would be the key to understanding how the system can be perturbed for increased potency^35^. In addition, a tunable switch system to control levels of gene therapy expression has been described, which could be used to optimize protein tissue levels^36^. These studies collectively suggest that gene therapy can result in sustained tissue protein expression. However, there are no previous studies that directly quantify human protein tissue levels after gene therapy in nonhuman primates. Therefore, the current study is the first to specifically quantify human tissue protein expression after gene therapy, providing insight into why this is so important because of the consequences of non-linear pharmacokinetics.

Preclinical gene therapy studies in animal models are required before the vectors can be tested in humans. Nonhuman primates offer significant advantages over rodent and canine models especially in models of rare genetic diseases^37^. Nonhuman primate studies are essential for the safety assessment of AAV-based gene therapy products prior to human studies^38^. They have also been very useful for assessing the neuropathology associated with gene therapy targeted at the central and peripheral nervous system^39^, and for assessing the immune response to helper dependent adenoviral mediated liver gene therapy^40^. Importantly, the expressed proteins from non-human primate are generally very similar in sequence to human proteins, which limits the potential immunogenicity of a foreign protein^38^. In the case of hFXN-M, there are only two amino acid differences when compared with mFXN-M, so the sequences are 98.5% identical (Fig. 1). In, contrast mouse FXN-M has 12 amino acid differences when compared with hFXN-M, so the sequences are only 90.8% identical^25^. Furthermore, mFXN-M is expressed as two major proteoforms in the mitochondria and cytosol of cells from mouse heart tissue, whereas hFXN-M is only found as a single proteoform in the mitochondria of cells from human heart tissue^25^. The advantage of using non-human primates for hFXN-M gene therapy studies presents a formidable bioanalytical challenge in quantification of proteins with almost identical sequences. One needs to be able to quantify precisely and distinguish both the endogenous non-human primate protein and the almost identical therapeutic gene product human protein. in the case of hFXN-M and mFXN-M, we were able to develop a novel method able to distinguish and quantify the two proteins using stable isotope dilution IP-LC-MRM/MS (Fig. 2), offering unprecedented insight in transgene product quantification using a species relevant protein for pharmacology and safety evaluation.

Previous gene therapy studies conducted in mouse models suggested that toxicity occurred through overexpression of FXN-M protein^18^, although the various mouse FXN-M and hFXN-M proteoforms^25^ could not be distinguished by the assay methodology that was used^25^. Paradoxically, the toxicity appeared to result from a decrease in iron-sulfur cluster complexes^41^, although the biological activity of FXN-M involves (in part) the assembly of these complexes^42^. The mean level of hFXN-M in five control (non-FRDA) human hearts was found to be 5.1 ± 2.7 ng/mg tissue in the present study (Fig. 4E). The mean level of mFXN-M in control monkey heart was 2.1 ± 0.4 ng/mg tissue (Fig. 4E). The lowest dose of AAV vector of 1.00E13 GC/kg resulted in the expression of hFXN-M at a mean level of 5.1 ± 1.7 ng/mg tissue (Fig. 7A) which is close to the endogenous level of hFXN-M in human heart and mFXN-M in monkey heart. In contrast, a three-fold increase in dose of the AAV vector (3.00E13 GC/kg) resulted in an almost 10-fold increase in the level of hFXN-M protein in heart tissues (Fig. 7B). Furthermore, heart tissue protein concentrations after a ten-fold dose increase (1.00E14 GC/kg were 20-fold higher than the endogenous levels of mFXN-M in the monkey heart (Fig. 7C). These potentially toxic concentrations with modest increases in the AAV vector dose illustrate the need for careful pharmacokinetic studies prior to using vector in clinical studies to treat patients. It is evident that mRNA data did not predict the observed proteins levels. In the present short-term 28 days study, no toxicity was observed although longer timepoints would be needed to address to possible toxicity of such hFXN-M levels on the long term.

On the positive side, our study, which has rigorously quantified both the human and monkey protein in a gene therapy study, has shown that the lowest dose of vector resulted in the expression of hFXN levels in monkey hearts at similar levels to those found in control (non-FRDA) human and monkey hearts. It is plausible that these levels of hFXN-M in human hearts would prevent the cardiomyopathy associated with Friedreich’s ataxia. Furthermore, the highly sensitive and specific stable isotope dilution IP-UHPLC-MRM/MS that was developed in the current study will allow hFXN-M to be quantified in only 5 mg human heart biopsy tissue taken before and after administration of the AAV vector to FRDA patients. Such studies are currently under way. Finally, the insights from the present study can be applied to all gene therapy studies, which will allow essential pharmacokinetic parameters to be determined in non-clinical models as well as in patients undergoing gene therapy, and to objectively compare expression levels achieved across different studies. We anticipate that this will simplify and expedite the selection of the most appropriate gene therapy dose to optimize efficacy and avoid the toxicity that can occur with overexpression of bioactive proteins.

## Methods

### Materials and reagents.

Protein G Dynabeads for immunoprecipitation, RIPA lysis buffer with EDTA, formic acid Optima LC/MS Grade, and CaptureSelect^™^ AAV9 resin were from ThermoFisher Scientific (Waltham, MA). Phosphate-buffered saline (PBS) ammonium bicarbonate (NH_4_HCO_3_), dimethyl pimelimidate dihydrochloride (DMP), ethanolamine, triethylamine (TEA), 0.2 M cross-linking buffer, pH 8.0, dithiothreitol (DTT), acetic acid (glacial), Tween-20, EDTA-free Easypack protease inhibitor cocktail tablets, bovine serum albumin (BSA), and sodium dodecyl sulfate (SDS) were from MilliporeSigma (Billerica, MA). Trypsin endoproteinase sequencing grade was from Promega (Madison, WI), acetonitrile (ACN), LC/MS grade water and Optima LC/MS grade, were from Fisher Scientific (Pittsburgh PA). Mouse anti-frataxin monoclonal antibody (mAb) Ab113691 raised against the N-terminal sequence of hFXN was from Abcam (Walton, MA). hFXN-M protein and SILAC-hFXN-M protein internal standard were prepared as previously described^43^. De-identified fresh frozen control (non-FRDA) heart tissue was supplied by BioIVT (Hicksville, NY) from consented donors and the relevant IRB approval was obtained. Subjects had typically died from heart failure.

### AAV Vector.

The Penn Vector Core produced and titrated AAV vectors for the study as previously described^44^. In brief, HEK293 cells were triple-transfected, and the culture supernatant was harvested, concentrated, and purified with an iodixanol gradient. The vector was produced by triple transfection of adherent HEK293 cells and purified from supernatant by affinity chromatography using a POROS^™^ CaptureSelect^™^ AAV9 resin, followed by anion exchange chromatography. Limulus amebocyte lysate and quantitative polymerase chain reaction (qPCR) tests for endotoxin and mycoplasma, respectively, were negative. Vector titer by TaqMan PCR was 6.05 × 10^13^ genome copies (GC)/mL. The purity of capsid proteins was 95.34%, as determined by sodium dodecyl sulfate-polyacrylamide gel electrophoresis analysis. Purified vectors were titrated with droplet digital PCR using primers targeting the rabbit beta-globin polyA sequence as previously described^45^.

### Animal Dosing.

The use Rhesus macaque (Macaca mulatta) monkeys in the study was approved by IUCAC of the University of Pennsylvania Approval #806620. The macaques were procured from Orient BioResource Center Inc. via PreLabs. Animals were 3.6–4.5 years old and weighed between 4.6–5.5 Kgs at study initiation. The animals were housed in the AAALAC International-accredited Nonhuman Primate Research Program facility at the University of Pennsylvania in stainless steel squeeze-back cages as groups. Animals received varied enrichments such as food treats, visual and auditory stimuli, manipulatives, and social interactions. The monkeys received a single intravenous injection (Saphenous vein) of the vector at 1 X10^13^, 3 X 10^13^ and 1X 10^14^ GC/Kg.

### Tissue samples.

At study day-28, animals were euthanized, and necropsies were performed. Hearts were removed from the animals’ and samples from ventricles, aorta, and septum were collected and immediately frozen to −80 °C and stored at this temperature until analyzed.

### Preparation of DMP crosslinked Dynabeads.

A Dynabead suspension (5 mg, 150 μL) was transferred to a Sarstedt 2.0 mL low bind (LB) microtube (Sarstedt, AG Numbrecht, Germany). and washed three times using 500 μL of bead washing buffer (PBS with 0.02% Tween-20). Mouse anti-FXN mAb Ab113691 (40 mg, 80 mL) was diluted using PBS to a final volume of 500 μL in a Sarstedt 2.0 mL LB microtube. The beads were incubated with the mouse mAb at 4 °C overnight on a Mini LabRoller rotator (Labnet International, Edison, NJ). The beads were swirled so that they were and thoroughly suspended, the mAb solution removed, and the beads washed twice with 1 mL of the 0.2 M TEA cross-linking buffer. A solution of DMP was prepared by dissolving 13 mg of DMP in 2 mL cross-linking buffer (freshly made every time). The beads were incubated with the 2 mL DMP solution at room temperature (RT) in a Sarstedt 2.0 mL LB microtube for on the rotator. After 1 h, the DMP solution was removed and the beads washed with 1 mL of quenching buffer (0.1 M ethanolamine, pH 8.0; 301 μL in 50 mL water). This was followed by incubation of the beads with 1 mL of quenching buffer at RT on the rotator for 1 h, removal of the quenching buffer and wishing the beads twice with 1 mL of bead washing buffer. DMP cross-linked beads were used immediately.

### IP of heart tissue samples.

DMP cross-linked beads were gently well-mixed in 1 mL of bead wash buffer then pipetted in 100 μL aliquots (0.5 mg beads/sample) into Sarstedt 2.0 mL LB microtubes. Monkey heart tissue samples (20 mg to 200 mg) were weighed and transferred to a Sarstedt 2.0 mL LB microtube. Wash and dry the tube, accurately re-weigh it and record the amount of heart tissue. RIPA lysis buffer (500 μL) containing the protease inhibitor cocktail was added to heart tissue followed by approximately, 30–50 stainless steel beads (0.9–2.0 mm). Homogenization was conducted using the Bullet Blender Gold homogenizer (Next Advance, Troy, NY) at a speed of 10 for 5 min at 4 °C. Samples were then lysed completely by additional probe sonication on ice with 30 pulses at power 4 using a sonic dismembranator (Fisher Scientific, Pittsburgh, PA). The SILAC-hFXN-M internal standard solution (20 μL of 2 μg/mL, 40 ng) was then added to each sample and appropriate amounts of hFXN-M standards ng to 200 ng (4, 10, 15, 20, 30, 40, 80, 100, 150, 200 ng) in 5% BSA solution for preparation of the standard curve^12^. Supernatants were removed from the DMP cross-linked beads and tissue and BSA standard samples added into the Sarstedt 2.0 mL LB microtubes containing the DMP cross-linked beads. Samples were then incubated with the beads at 4 °C overnight on the rotator.

### Elution of hFXN-M and mFXN-M from the beads.

Supernatants with unbound proteins were removed and the beads re-suspended in bead wash buffer (1 mL). The beads were washed twice with bead wash buffer (1 mL) then transferred to clean 1.5 mL microcentrifuge tubes (ThermoFisher Scientific, Waltham, MA). After washing the beads with PBS (1 mL), hFXN-M and mFXN-M were eluted with 200 μL of elution buffer (100 mM acetic acid in 10% ACN) by shaking the beads in a thermal mixer (ThermoFisher Scientific, Waltham, MA) at 1000 rpm and RT for 1 h. Supernatants were then transferred to Sarstedt 2.0 mL LB microtubes and dried under a nitrogen flow using an N-Evap concentrator (Organomation, Berlin, MA).

### Trypsin digestion of eluted hFXN-M and mFXN-M.

Dried eluates from the Dynabeads were dissolved in 50 mM NH_4_HCO_3_ solution (50 μL) containing trypsin (100 ng). Samples were Incubated at 37 °C for 18 h then centrifuged at 15,000g for 2 min on a bench top centrifuge (Eppendorf North America, Enfield, CT) at RT. Supernatants were transferred to Waters 150 μL insert tubes with pre-installed plastic springs (Waters, Milford, MA), which were then placed into Waters LC-MS certified PTFE/silica 2 mL screw-top injection vials ready for analysis by UHPLC-MRM/MS.

### UHPLC-MRM/MS analysis.

Solutions (2 μL) containing the tryptic peptides were injected in triplicate on the UHPLC-MRM/MS system. This was equivalent to the injection of a trypsin digest from heart tissue spiked with 1.6 ng (105 fmol) of SILAC-hFXN-M protein internal standard. An Agilent 1290 Infinity UHPLC system equipped with a Zorbax Rapid Resolution High Definition (2.1 × 50 mm, 1.8 μm particle size) UHPLC column was used. It was coupled on-line to an Agilent 6495C triple quadruple mass spectrometer. Mobile solvent A was 0.1% formic acid in water and solvent B was 0.1% formic acid in ACN. The UHPLC column at was maintained 35 °C with a flow rate of 0.4 mL/min. The tryptic peptides were separated with the following linear gradient: 5% B at 0-min, 5% B at 1-min, 24% B at 2.75-min, 36% B at 3.50-min, 95% B at 5.0-min, 95% B at 6.50-min, 5% B at 7.0-min, 5% B at 8.5-min ready for the next injection. Mass spectrometer operating conditions were maintained as follows: nitrogen gas flow 13 L/min, gas temperature 230 °C, nebulizer gas 40 psi, sheath gas temperature 300 °C, sheath gas flow 10 L/min, capillary voltage 4500 V, and nozzle voltage 500 V. The MRM/MS transitions shown in Table 1 were used and care was taken to ensure that the retention times (ret time) were within 0.1 min of the times shown in Table 1.

### Data analysis.

Peptide quantification was performed using Skyline (MacCoss Laboratory, University of Washington, Seattle, WA)^46^. The peak area ratio of each MRM transition for each unlabeled/light (L) peptide to labeled/heavy (H) peptide was calculated by the Skyline software and used for absolute quantification. The peptide ratios were calculated from the sum of L/H ratios of the MRM transitions of the y142+-, y5+-, and y3+-ions specific for SGTLGHPGSLD**E**TTYER the N-terminal tryptic peptide specific for hFXN-M (blue signifies the amino acid specific to hFXN) and y142+-, y5+-, and y4+-ions for the heavy SGTLGHPGSLD**E**TTYER internal standard as well as and y193+-, y8+-, and y7+-ions from NWVYSHDGVSLHELL**A**AELTK tryptic peptide specific for hFXN-M and y193+-, y8+-, and y7+-ions from the heavy NWVYSHDGVSLHELL**A**AELTK internal standard (lower). The peptide ratios were then used to calculate the mean amount of hFXN-M from the relevant standard curves (Figs. 2B and 2C). Similarly, the peptide ratios were calculated from the sum of L/H ratios of the MRM transitions of the y142+-, y5+-, and y_3_^+^-ions of the SGTLGHPGSLD**D**TTYER N-terminal tryptic peptide from mFXN-M (red signifies the amino acid specific to mFXN) and y142+-, y5+-, and y4+-ions from the heavy SGTLGHPGSLD**E**TTYER internal standard as well as the y193+-, y8+-, and y7+-ions from NWVYSHDGVSLHELL**G**AELTK tryptic peptide from mFXN-M and y193+-, y8+-, and y7+-ions from the heavy NWVYSHDGVSLHELL**A**AELTK internal standard. The peptide ratios were then used to calculate the mean amount of mFXN-M from the relevant standard curves (Figs. 2B and 2C). Standard curve for this peptide constructed using triplicate injections of known amounts of hFXN-M in the range 4 ng to 200 ng (4, 10, 15, 20, 30, 40, 80, 100, 150, 200 ng) with 40 ng of SILAC-hFXN-M as internal standard extracted by IP from BSA. The slope, intercept, coefficient of determination (r^2^), the back-calculated accuracy and the precision (%CV) for the standard curves were then determined for each peptide. Results for heart tissue samples are reported as ng/mg tissue using the average of three replicates. Three MRMMS transitions for three other tryptic peptides common to both FXN-M and mFXN-M (DKPTYFE and DLSSLAYSGK) were monitored to provide additional confirmation that FXN-M was present.

## Data Availability

The data that support the findings of this study are available within the paper. Any additional information not included in the paper is available upon request from Dr. Ian. A. Blair or Dr. James M. Wilson.
